# Laboratory investigation of the factors impact on bubble size, pore blocking and enhanced oil recovery with aqueous Colloidal Gas Aphron

**DOI:** 10.1007/s13202-015-0193-7

**Published:** 2015-08-15

**Authors:** Shenglong Shi, Yefei Wang, Zhongpeng Li, Qingguo Chen, Zenghao Zhao

**Affiliations:** School of Petroleum Engineering, China University of Petroleum (East China), Qingdao, People’s Republic of China

**Keywords:** Colloidal Gas Aphron, Bubble growth rate, Block, Enhanced oil recovery

## Abstract

Colloidal Gas Aphron as a mobility control in enhanced oil recovery is becoming attractive; it is also designed to block porous media with micro-bubbles. In this paper, the effects of surfactant concentration, polymer concentration, temperature and salinity on the bubble size of the Colloidal Gas Aphron were studied. Effects of injection rates, Colloidal Gas Aphron fluid composition, heterogeneity of reservoir on the resistance to the flow of Colloidal Gas Aphron fluid through porous media were investigated. Effects of Colloidal Gas Aphron fluid composition and temperature on residual oil recovery were also studied. The results showed that bubble growth rate decreased with increasing surfactant concentration, polymer concentration, and decreasing temperature, while it decreased and then increased slightly with increasing salinity. The obvious increase of injection pressure was observed as more Colloidal Gas Aphron fluid was injected, indicating that Colloidal Gas Aphron could block the pore media effectively. The effectiveness of the best blend obtained through homogeneous sandpack flood tests was modestly improved in the heterogeneous sandpack. The tertiary oil recovery increased 26.8 % by Colloidal Gas Aphron fluid as compared to 20.3 % by XG solution when chemical solution of 1 PV was injected into the sandpack. The maximum injected pressure of Colloidal Gas Aphron fluid was about three times that of the XG solution. As the temperature increased, the Colloidal Gas Aphron fluid became less stable; the maximum injection pressure and tertiary oil recovery of Colloidal Gas Aphron fluid decreased.

## Introduction

A kind of micro-bubbles system with a special structure named Colloidal Gas Aphron (CGA) is prepared by stirring a surfactant-xanthan gum solution at high speeds (above 4000 rpm, for example). The average bubble diameter of CGA can be about 10–100 μm; the behavior is that of a colloidal dispersion of a gas in a liquid (Ivan et al. 2002). Unlike ordinary foam bubbles, CGA has a unique thin aqueous protective shell. The CGA is a gaseous inner core encapsulated by an inner and outer surfactant shell. There is a viscous water layer located between these two surfactant layers, and the viscous water layer is important to CGA stability. A stable CGA structure requires keeping a film thickness of 4–10 μm^2^. The film can decrease the transfer rate of the surfactant molecules between the viscous water layer and the bulk phase. The second performance of CGA is low diffusivity, which is the ability of the air that is in the core to transfer to the aqueous shell (Bjorndalen and Kuru 2008).

Brookey first recommended aqueous CGA as a novel drilling fluid to the petroleum industry. He wrote about the potential of using CGA in drilling fluids to reduce near wellbore formation damage (Brookey 1998). CGA has also been studied to enhance blocking capacity. Growcock et al. (2006) investigated the flow of CGA in porous media by microcosmic visualization model; they summarized that when CGA drilling fluid entered the formation, the CGA moved forward rapidly to concentrate at the fluid front and established a microenvironment that segregated the bulk fluid from the formation. There was successful blockage of the micromodel and porous media by the stable CGA fluid as compared to the flow of fluids formulated with only polymer and only surfactant; pressure drop through porous media increased continuously as more CGA fluid was injected into the porous media (Bjorndalen et al. 2014). The CGA fluids showed more stable frontal displacement, lower injection pressure and longer retention time as compared to polymer flooding (Samuel et al. 2012). These characteristics of the flow behavior of CGA in porous media were the main motivation behind the idea of using CGA for applications in enhanced oil recovery. However, the actual reservoir heterogeneity caused by water channeling should be taken into account in laboratory experiments.

The purpose of this investigation is to evaluate technical feasibility of improving oil recovery and the extent of resistance flow through porous media of CGA fluid. Change in the size of the micro-bubbles, apparent viscosity and low shear rate viscosity (LSRV) of the CGA were recorded as a measure of the stability. The effect of surfactant concentration, polymer concentration, temperature and salinity on the CGA bubble size was investigated. The effects of the CGA formulations and formation heterogeneity on pressure drop were examined by CGA fluid injection. The effects of CGA formulations and temperature on the performance of CGA fluid on oil recovery were also studied.

## Equipment and materials

### Materials

The chemical agents used in this study included polymer and surfactant. The polymer was xanthan gum with a molecular weight of 3.4 × 10^6^ (XG) provided by Changxing Chemical Company, China, and the surfactant was hydroxyl sulfobetaine (SL1) provided by Shengli Oilfield, with a purity of 33 %; the water used in experiments was tap water or simulated formation water. The simulated formation water consisted of sodium chloride and calcium chloride; the total dissolved solid value of the simulated formation water ranged from 20,000 to 160,000 mg/L and the mass ratio of Na^+^ and Ca^2+^ was 19:1. Oil was collected from Changqing Oilfield in China; the oil had a viscosity of 15.5 mPa s and a density of 0.835 g/cm^3^ at 20 °C. The acid number value of the oil was analyzed to be 0.21 mg KOH/g of sample. All of these tests were conducted at the 20 °C, except where otherwise specified.

### CGA fluid formation and bubble diameter change with time

The base fluid was prepared as XG and SL1 in tap water, and then the base fluid was stirred at 3500 rpm for 2 min by a blender (GJ-3S, Qingdao Senxin Machinery Equipment Co., Ltd., China); the initial foam volume and the time required for the foam to drain one-half of initial volume solution were recorded. The formed CGA was also observed using a microscope (BM 1000, Nanjing Jiangnan Optical Co., Ltd., China). Microscopic pictures were taken at 10 min intervals depending on the formulation of the CGA fluid. The diameters of the CGA bubbles were determined by measuring the sizes of at least 200 bubbles from recorded pictures using custom-made image-analysis software.

### Measurements of apparent viscosity and low shear rate viscosity

The apparent viscosity of the fluid was measured by using the Brookfield DV II Digital Cone Viscometer (Brookfield, America). The LSRV of CGA was measured at the shear rate of 0.1 s^−1^ by MCR 302 coaxial cylinder rheometer (Andon Paar, Austria).

### CGA fluid injection

**Fig. 1:**
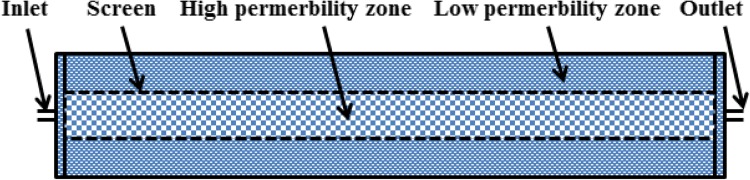
Schematic of heterogeneous sandpack

The homogeneous and heterogeneous sandpacks used for tests were 2.5 cm in diameter and 20 cm in length. There was a screen with a 1.0 cm diameter, which was placed at the center of the heterogeneous sandpack. The coarse sand was packed in the channel and the fine sand was packed in the annulus between the screen and the inner wall. The screen allows the communication of the fluid between the high and low permeable zones during the injection process. The schematic diagram of the heterogeneous sandpack is shown in Fig. 1. The sandpack was saturated with tap water; the permeability and injection porous volume (PV) of the sandpack were measured. The CGA fluid was injected at a fixed injection rate into the sandpack until the maximum pressure was obtained. Once this point was reached, the CGA injection was stopped and tap water was re-injected at the same rate until equilibrium pressure was reached. The CGA fluid was prepared in tap water.

### CGA fluid for oil recovery

The homogeneous sandpacks used for tests were 2.5 cm in diameter and 40 cm in length. The experimental procedure was as follows: First, the sandpack was saturated with simulated formation water; the permeability and porous volume of the sandpack were measured; crude oil of 2 PV was injected into the sandpack followed by a 72 h aging stage at 20 °C. After the initial oil saturation was set, the simulated formation water was continued until the oil production became negligible (oil cut less than 2 %). After the water flooding, 1 PV of CGA fluid or 1 PV of XG solution was injected to compare their performances as recovery fluids, followed by extended water flooding until the oil production became negligible. The data collected from the sandpack flood tests consisted of produced fluid volumes and pressures. The tests were conducted at 20 to 60 °C. The water, CGA fluid and XG solution were injected at a rate of 2 mL/min and they were prepared in 120,000 mg/L simulated formation water.

## Results and discussion

### Measurement of CGA bubble diameter with time

**Fig. 2:**
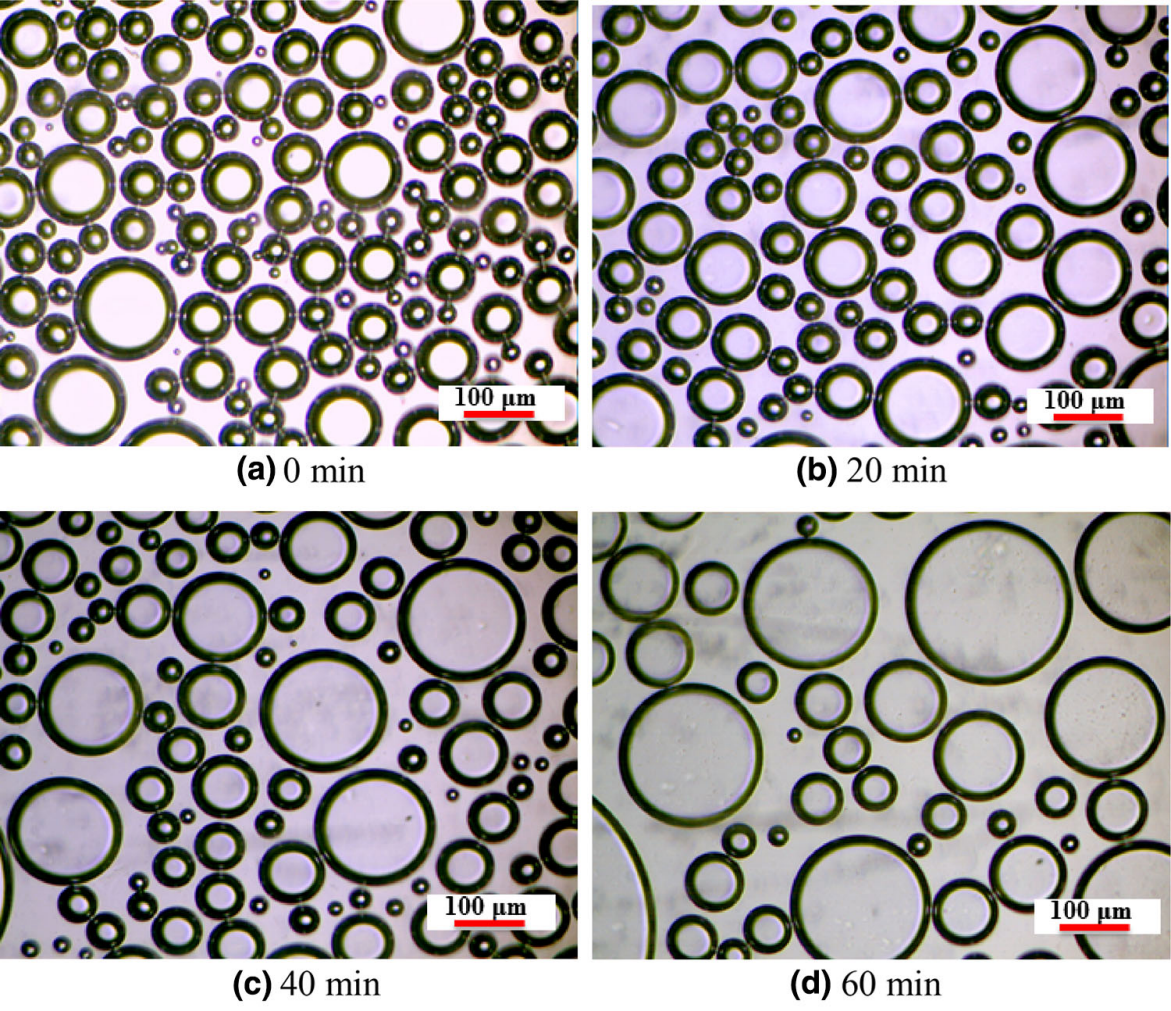
Microscopic images taken of CGA bubble size with time

The increase of the CGA bubble with time is an important element for determining the ability of the bubble to block pore media. Figure 2 shows an example of the microscopic images taken for analysis of bubble diameter with time. CGA possessed a strong, impermeable shell, bubbles existed in separate spheres, and there was no plateau border between bubbles. The volume of bubbles grew bigger, liquid film became thinner and number of bubbles decreased as a function of standing time.

#### Effect of surfactant concentration

**Fig. 3:**
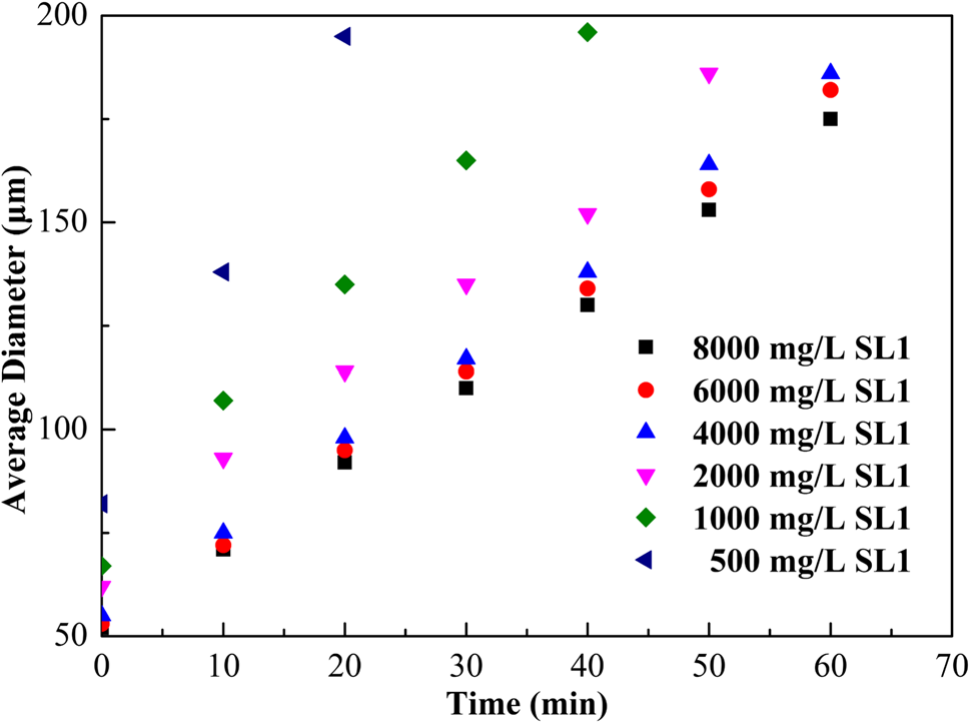
Effect of SL1 concentration with 6000 mg/L XG on the average diameter of the CGA bubble

The effect of surfactant concentration on increasing in average bubble size with time is shown in Fig. 3. CGA bubble grew bigger as a function of time. The rate of bubble growth, that is to say, bubble deformation rate as a function of time decreased with increasing surfactant concentration. Increasing SL1 concentration decreased the initial bubble size. The initial bubble sizes of the CGA solutions were 55–80 µm, respectively. When SL1 concentration was over 4000 mg/L, the variation of bubble size with time was slight. This implied that the increase of SL1 concentration above 4000 mg/L did not seem to have any effect on the size of the bubbles.

#### Effect of polymer concentration

**Fig. 4:**
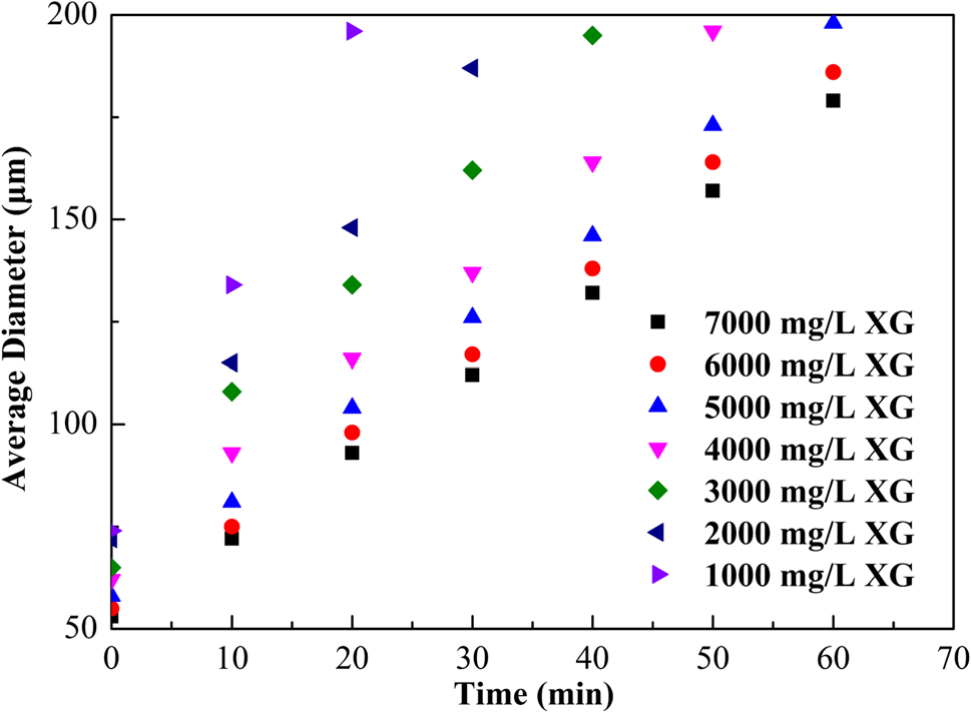
Effect of XG concentration with 4000 mg/L SL1 on the average diameter of the CGA bubble

**Fig. 5:**
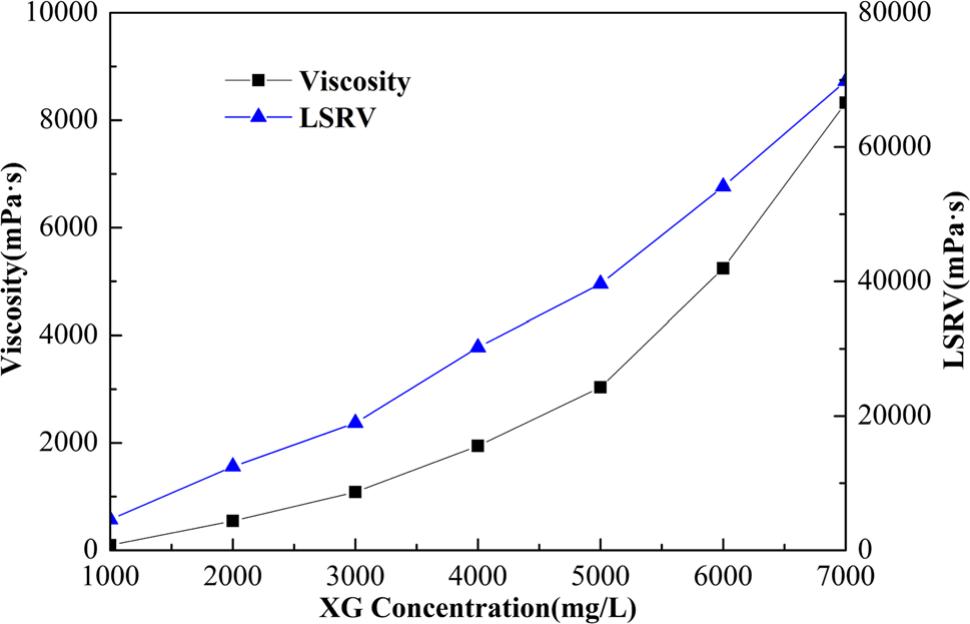
Effect of XG concentration with 4000 mg/L SL1 on apparent viscosity and LSRV of CGA fluid

Figure 4 depicts the experimental results for the change in bubble size with time for different XG concentrations. The CGA bubble became unstable as a function of time. The degree of bubble expansion with time decreased as XG concentration increased, thus confirming that an increase in viscosity of the base fluid increased the stability of the CGA system. Also Fig. 5 shows that the initial average bubble diameter was similar at approximately 60 µm for all XG concentrations. For the 6000 mg/L XG sample, it took about four times longer to reach the same bubble size (160 µm) than it did for the 2000 mg/L XG sample. Furthermore, a change in XG concentration from 6000 to 7000 mg/L had little effect on the change of bubble size with time. This indicated that increasing the XG concentration above 6000 mg/L did not have a significant impact on inhibiting the CGA bubble expansion.

Figure 5 shows the effect of XG concentration on apparent viscosity and LSRV of CGA fluid. Remarkable increase in apparent viscosity and LSRV was observed for CGA system with increased XG viscosity. If fluid LSRV is lowered below 40,000 mP s, the CGA would become unstable and break apart. XG at a concentration of 6000 mg/L was chosen as its LSRV was above the critical value of 40,000 mP s (MacPhail et al. 2008).

#### Effect of temperature and salinity

**Fig. 6:**
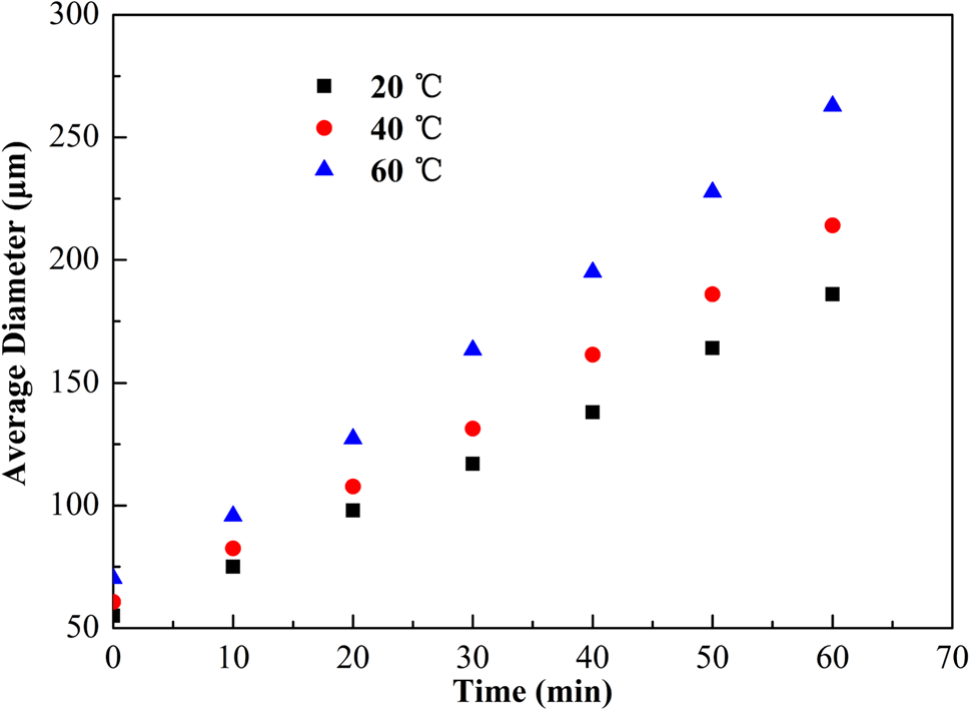
Effect of temperature with 4000 mg/L SL1 and 6000 mg/L XG on the average diameter of the CGA bubble

Figure 6 shows the change in bubble size as a function of time with 4000 mg/L SL1 and 6000 mg/L XG at base fluid temperature of 20, 40 and 60 °C, the base fluid was prepared in tap water. Increasing the base fluid temperature had an obvious impact on the change of bubble size with time. Between 40 and 60 °C, bubble growth rate changed at a faster rate than that of the temperature from 20 to 40 °C, indicating that CGA fluid seem to be more sensitive to temperature change at higher temperatures. This was mainly because that high temperature accelerated gas diffusion velocity and liquid film drainage rate, which would result in decreasing viscosity and thickness of shell. As a result, the resistance to bubble growth was decreased and bubble growth rate was increased. Therefore, an increase of temperature resulted in a decline in CGA fluid stability.

**Fig. 7:**
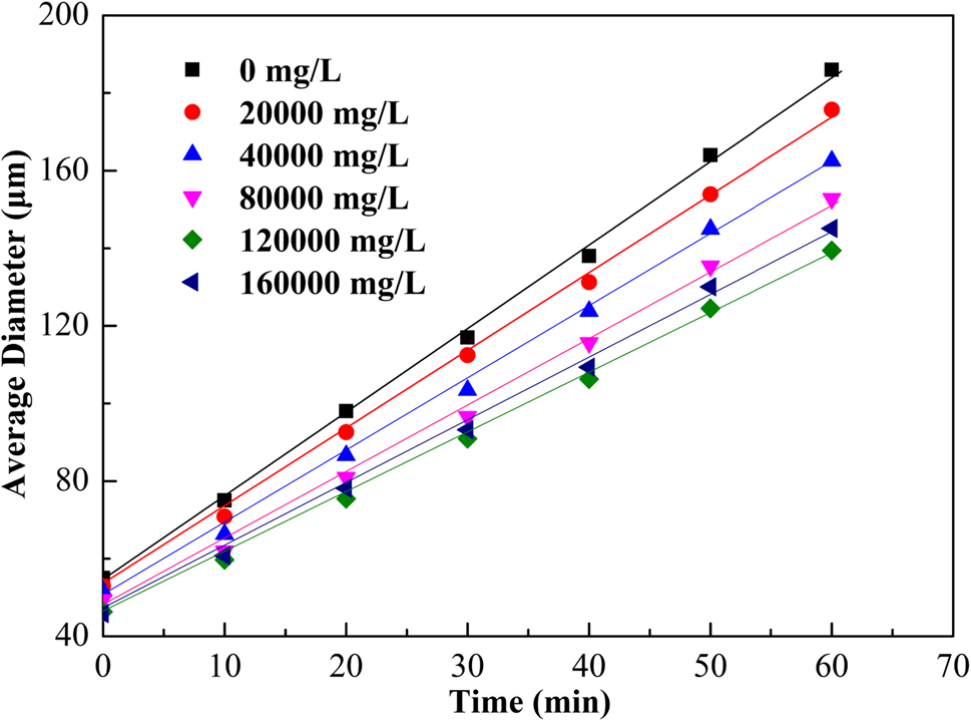
Effect of salinity with 4000 mg/L SL1 and 6000 mg/L XG on the average diameter of the CGA bubble

The change in bubble size of CGA fluid with time was studied at different salinity levels and the result is shown in Fig. 7; the tested temperature was 20 °C with 4000 mg/L SL1 and 6000 mg/L XG. It could be seen that bubble growth rate declined and then increased slightly with increasing salinity; the bubble growth rate was in the range from 2.18 to 1.55 μm/min, and the salinity ranged from 20,000 to 160,000 mg/L, and the bubble growth rate reached the lowest value with salinity of 120,000 mg/L. The results showed that the CGA fluid could be applied in a very wide range of salinity, and it could be put into use and promoted in high-salinity reservoirs.

### CGA fluid injection

The CGA fluid injection tests were investigated at different injection rates, surfactant concentrations, polymer concentrations, and reservoir heterogeneity. Table 1 lists the parameters used for those tests.

**Table 1:** Parameters used for CGA fluid injection tests

Experiment	Porosity/%	Permeability/μm^2^	XG concentration/(mg/L)	SL1 concentration/(mg/L)	Flow rate/(mL/min)
Ho1	34.29	1.04	1000	4000	2
Ho2	33.82	0.99	2000	4000	2
Ho3	33.61	0.98	4000	4000	2
Ho4	33.48	0.97	6000	4000	2
Ho5	34.09	1.02	6000	0	2
Ho6	33.95	1.00	6000	2000	2
Ho7	32.94	0.95	6000	4000	3
He1	36.27	1.23	6000	4000	2

*Ho* homogeneous, *He* heterogeneous

#### Effect of injection rate

**Fig. 8:**
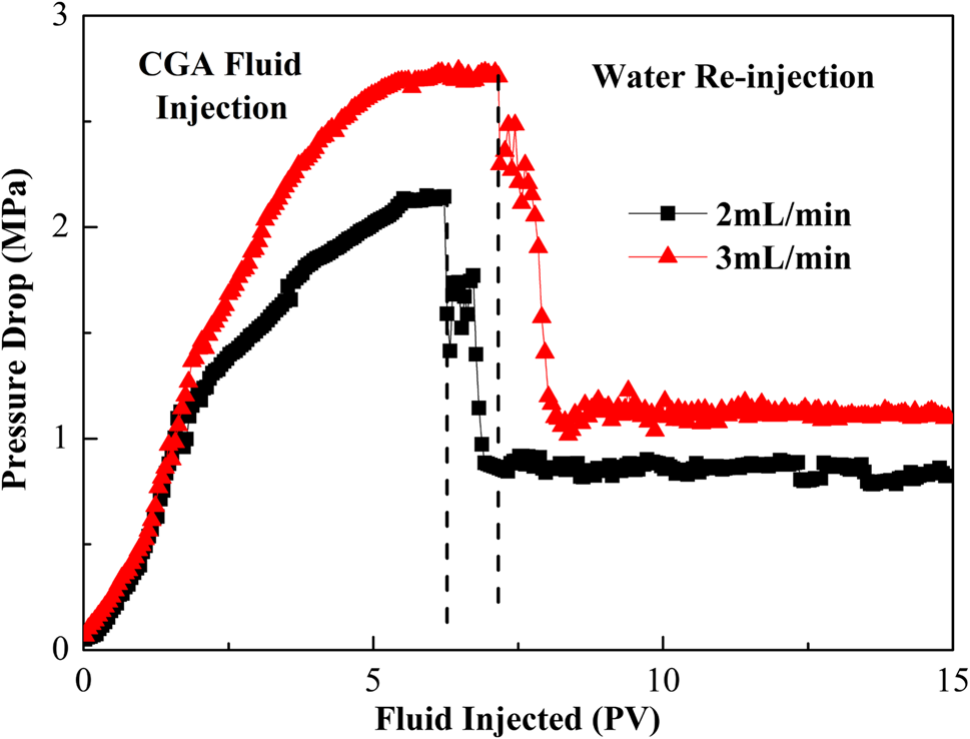
Effect of changing CGA fluid injection rate on the pressure drop

Figure 8 shows the effect of injection rate on the pressure drop across the sandpack. Fluid was injected at rates of 2 and 3 mL/min with corresponding shear rate across the radial cell varying from 10 to 100 s^−1^. This shear rate range represents the flow conditions experienced in water flooding experiments in typical reservoirs (Samuel et al. 2012). The continuous increase in pressure along the CGA injection indicated that the CGA was blocking the pores and throats of the porous media. When the injection rate was increased from 2 to 3 mL/min, the corresponding maximum injection pressure increased from 2.15 to 2.73 MPa. This indicated more effective pore blocking with the higher injection rate. Once switched to water, the CGA fluid was flushed out of the sandpack, and the pressure drop then decreased. When subsequent water was injected continuously, the pressure drop was still maintained at a value for more than the initial water-injection pressure level, indicating that the CGA fluid had a large resistance to water flushing.

#### Effect of surfactant concentration

**Fig. 9:**
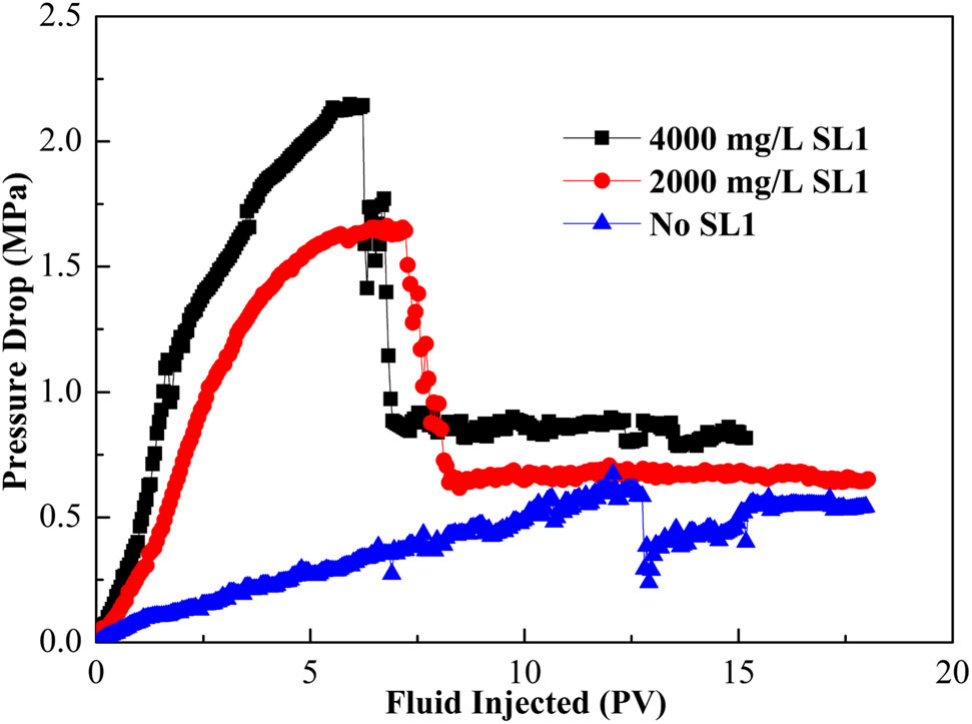
Effect of SL1 concentration with 6000 mg/L XG on the pressure drop

The effect of SL1 concentration on the pressure drop is shown in Fig. 9. The maximum pressure drop decreased as the surfactant concentration decreased with a base fluid of 6000 mg/L XG concentration. The case with 0 mg/L of SL1 contained no CGA bubbles and the case with 2000 mg/L of SL1 contained fewer CGA bubbles and stability of CGA was relatively weak; the total quantity of CGA bubbles flowing into the sandpack was not significant, so the pressure rise was much less. As the surfactant concentration increased, there were more micro-bubbles generated in the base fluid, a higher pressure drop would emerge. Therefore, it was important to have a surfactant concentration high enough (greater than 4000 mg/L) to create a stable CGA fluid.

#### Effect of polymer concentration

**Fig. 10:**
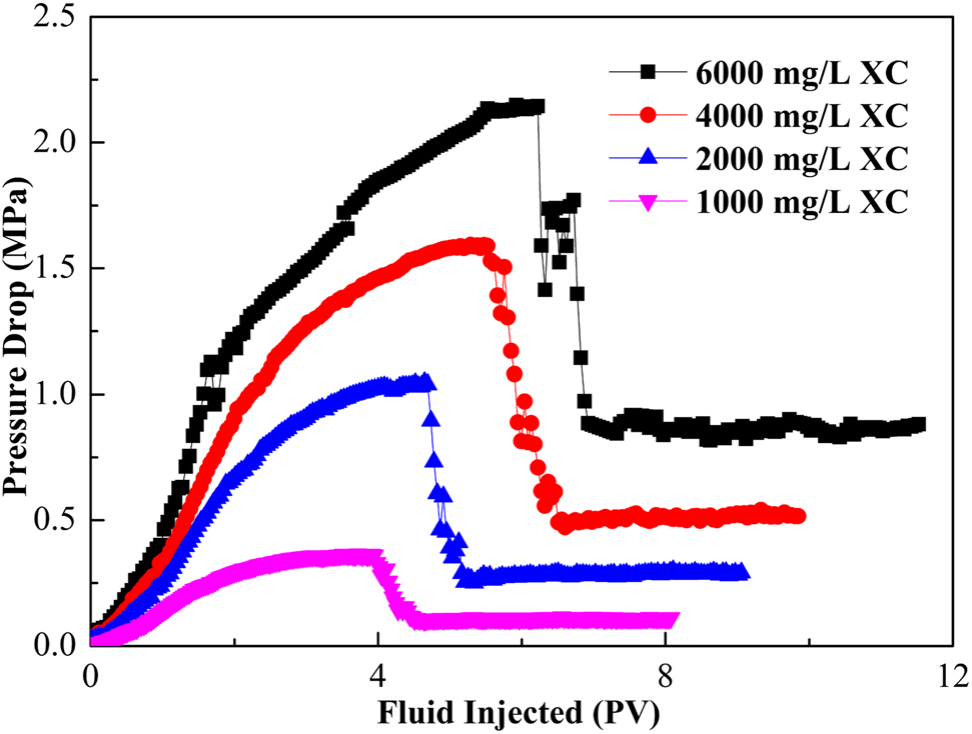
Effect of XG concentration with 4000 mg/L SL1 on the pressure drop

The change in pressure drop caused by the injection of CGA fluid with 4000 mg/L surfactant concentration and different XG concentration is shown in Fig. 10. As the polymer concentration increased, the pressure drop increased with CGA fluid injected. The effect was very obvious from 1000 to 6000 mg/L XG concentrations,which showed the more viscous and more stable the injection fluid, the greater pressure drop. The 1000 mg/L XG concentration was not as stable as the other XG concentration, the pressure rise was slightly less, and the experiment was terminated because of coalescence before 4 PV. This indicated that it was not viable to use a XG concentration of 1000 mg/L or less.

The stability of the CGA fluid decreased with the XG concentration decreased. It was, therefore, significant to have a XG concentration greater than 1000 mg/L to form a stable CGA fluid for an extended period of time. However, the greater the XG concentration brought about base solution with higher viscosity, the more difficult it became to generate stable CGA. Therefore, it was vital to determine a critical XG concentration that would give the optimum CGA formulation. For the system used in this study, the maximum XG concentration was 6000 mg/L and the minimum XG concentration was 1000 mg/L.

#### Effect of heterogeneity

**Fig. 11:**
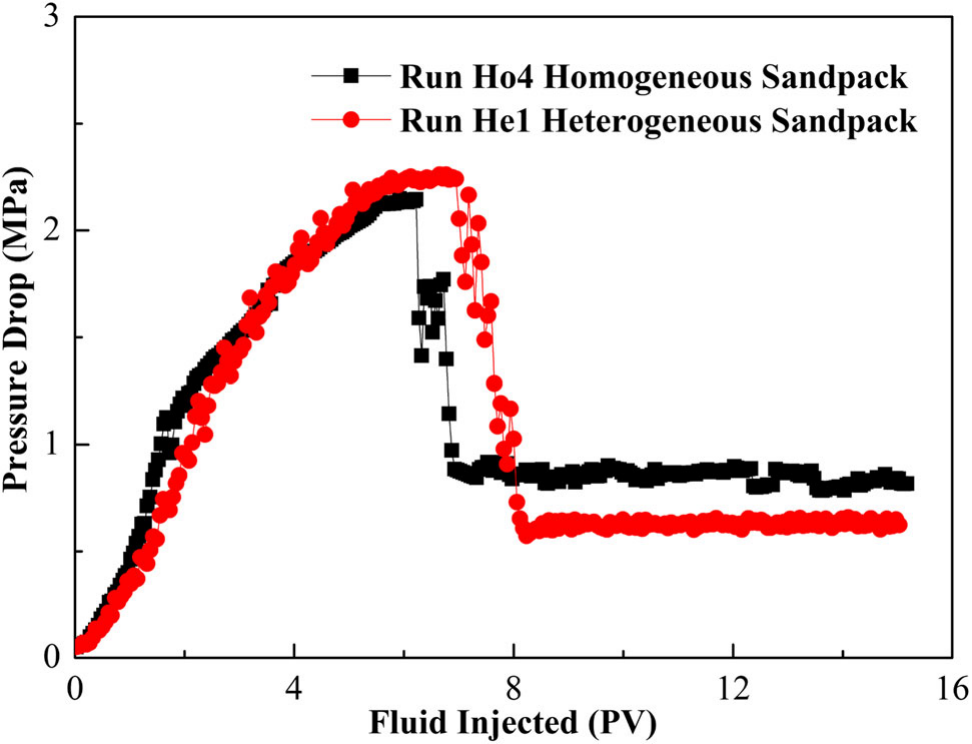
Comparison of pressure drop of experiment Ho4 and He1 as a function of pore volume of fluid injection

Using the chemical blend 6000 mg/L XG + 4000 mg/L SL1 to generate CGA, a heterogeneous sandpack displacement test, He1, was conducted to examine the effectiveness of this blend. The pressure drop curves both Ho4 and He1 as a function of pore volume of the injection fluid are shown in Fig. 11. Test He1 had a higher maximum injection pressure and stronger pressure fluctuation compared with that of test Ho4 during the CGA fluid injection. This indicated that CGA fluid would flow in zone of higher permeability preferentially and increased local resistance to flow, thereby diverting injected fluids to lower permeability zone that were previously inaccessible to injected water incapable of building up such pressure gradients. Higher local pressure gradients meant that more pores’ capillary entry pressures were exceeded, allowing fluid to mobilize and improving sweep efficiency. This phenomenon helped to explain how CGA fluid could flow in low permeability area adjacent to high permeability area.

The designed sandpack with a screen enables the study of the impact of heterogeneities on pressure drop in CGA fluid injection process. Even the injection process without any oil, the experimental result would also explain effectiveness of CGA for blocking the porous media in heterogeneous reservoirs.

### CGA fluid for oil recovery

To further assess the performance of the CGA fluid as an enhanced oil recovery technique, four sandpack flood tests were conducted, and efficiency of oil recovery using the CGA fluid was compared to that of the XG solution. Using the chemical blend 6000 mg/L XG + 4000 mg/L SL1 to generate CGA, XG solution was prepared using the same procedure with 6000 mg/L XG. Table 2 summarizes the results of CGA fluid and XG solution for oil recovery.

**Table 2:** Summary of the experimental results of CGA fluid and XG solution for oil recovery

Test	Porosity/%	Permeability/μm^2^	Initial oil saturation/%	Fluid composition	Temperature/ °C	Water flooding recovery/%	Tertiary recovery/%	Final recovery/%
1	31.67	1.28	85.68	CGA fluid	20	49.5	26.8	76.3
2	31.47	1.25	85.42	CGA fluid	40	52.3	23.5	75.8
3	30.86	1.23	84.49	CGA fluid	60	53.8	21.1	74.9
4	31.03	1.23	83.88	XG solution	20	47.8	20.3	68.1

#### Effect of fluid composition

**Fig. 12:**
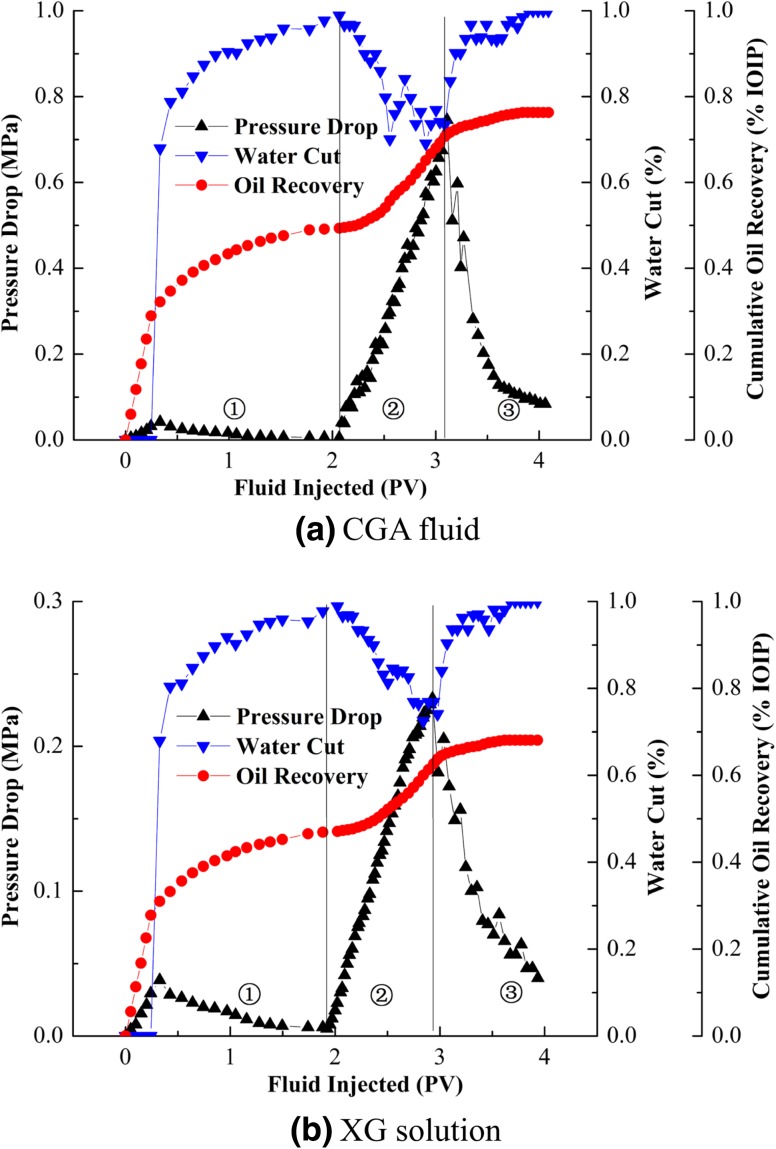
Results of sandpack flooding tests of CGA fluid or XG solution for oil recovery. ①-Water flooding ②-CGA flooding ③-Subsequent water flooding

Figure 12 compares the results of flooding test with CGA fluid and XG solution. It was observed that the injection pressure built up a first peak quickly and decreased slowly during water flooding, indicating the water breakthrough along the sandpack. After 2.0 PV of water was injected, the oil recovery was about 50 % and value of water cut reached 99 %. At this time, 1.0 PV of CGA was injected and the result is shown in Fig. 12a. There was increase in the pressure with fluctuation as the CGA fluid entered the pore spaces; this was because CGA fluid was not stable for contacting with oil at the beginning of injection and more prone to coalescence. It was observed that a sharp increase in pressure drop appeared during CGA fluid injection process. The sharp pressure drop indicated that the resistance of injected CGA fluid increased significantly, which diverting subsequent fluid contacted oil rich regions to improve sweep efficiency. When the injection pore volume was 1.0 PV, the highest injection pressure drop appeared, the value of lowest water cut reached 68.5 %. Once the solution was switched to water, the CGA fluid was flushed out of sandpack and the pressure drop then declined. Finally, CGA fluid enhanced oil recovery by 26.8 % and the total recovery could reach 76.3 %. This result indicated that CGA fluid had excellent displacement performance.

Figure 12b shows a smooth linear increase in pressure for the XG solution after water flooding, a slow buildup of XG solution occurred as more XG solution moved into and adsorbed the pores and throats which resulted in an increase in pressure. XG solution had a maximum injection pressure of 0.242 MPa, which was approximately one-third of that of the CGA fluid. This quite large pressure difference would favor the use of the CGA fluid as a recovery fluid in preference to the XG solution.

#### Effect of temperature

**Fig. 13:**
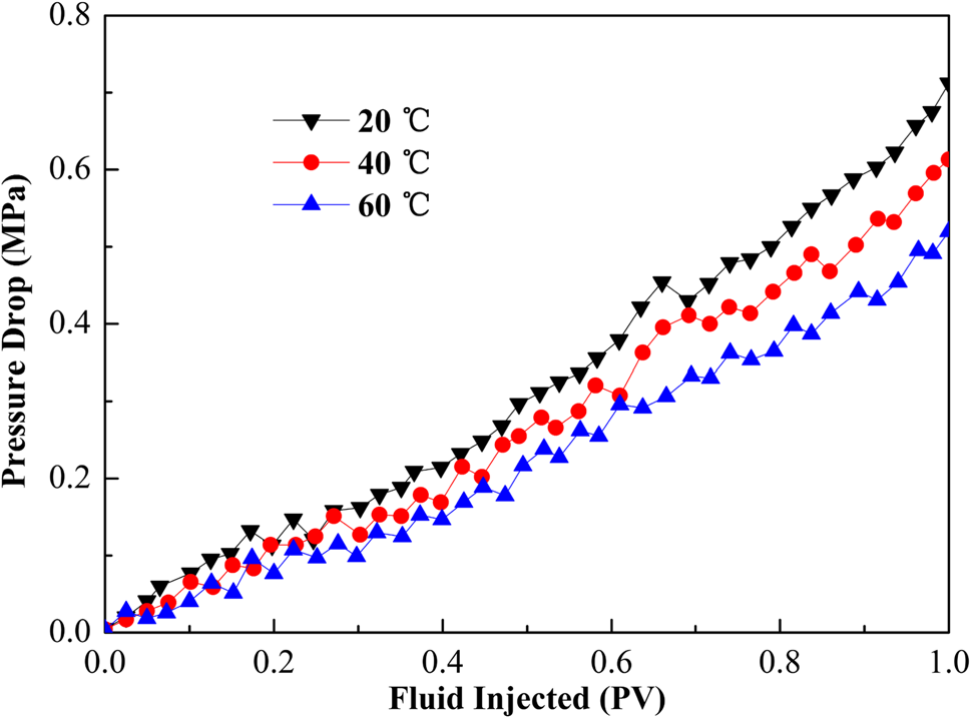
Effect of temperature on pressure drop as a function of CGA fluid injected

The results displayed in Table 2 show that the oil recovery by water flooding decreased with temperature from 20 to 60 °C. The viscosity of the crude oil was 15.5 mPa s at 20 °C, 8.1 mPa s at 40 °C, and 5.9 mPa s at 60 °C. The mobility ratio between water and oil decreased as temperature raise, resulting in an increase in oil recovery. Table 2 also showed that the oil recovery by CGA fluid decreased with temperature increase from 20 to 60 °C. For example, oil recovery was 26.8 % at 20 °C, 23.5 % at 40 °C, and 21.1 % at 60 °C. This decrease could be explained by the stability of CGA fluid lowered with increased temperature. As the temperature increased, the CGA fluid became less stable and the CGA fluid swept efficiency decreased. Furthermore, pressure drop responsed as a function of CGA fluid injected in the sandpack flooding tests at different temperatures were shown in Fig. 13. The decrease in peak value of pressure drop was accompanied by an increase in temperature, and a higher peak value of pressure drop resulted in a higher tertiary oil recovery. These results corresponded to that the sweep efficiency decreased with the temperature.

## Conclusions

As XG or surfactant concentration increased, initial average bubble diameter and bubble growth rate with time decreased. When XG concentration was above 6000 mg/L, or surfactant concentration was above 4000 mg/L, it did not have any significant effect on retarding the bubble growth rate and initial average bubble diameter. Between 40 and 60 °C, bubble sizes of CGA fluid changed at a faster rate than that of the temperature interval from 20 to 40 °C. While the salinity was in the range 0–160,000 mg/L, bubble growth rate decreased and then increased slightly with increasing salinity.

A high-pressure drop through the sandpack was observed when injecting CGA fluid continuously, which indicated that porous media were effectively blocked by stable CGA fluid. When XG or surfactant concentration decreased, the quantity and flow resistance of CGA in the system decreased. For the most effective CGA blocking, an XG concentration greater than 1000 mg/L but less than 6000 mg/L and a surfactant concentration of 4000 mg/L or greater, was observed. The effectiveness of the chemical blend selected through the homogeneous sandpack flood tests was modestly improved in the heterogeneous sandpack. The high permeability channel zone was blocked, and the low permeability zone obtained a further use because of increased pressure drop in the course of CGA fluid injection process. CGA fluid was found to improve oil recovery after water flooding in sandpack. The tertiary oil recovery increased 26.8 % by CGA fluid as compared to 20.3 % by XG solution when chemical solution of 1 PV was injected into the sandpack. The maximum injected pressure of CGA fluid was about three times that of the XG solution. The tertiary oil recovery by CGA fluid decreased from 26.8 to 21.1 % when temperature increased from 20 to 60 °C.

## Abbreviations

CGA: Colloidal Gas Aphron
LSRV: Low shear rate viscosity
XG: Polymer, xanthan gum, with a molecular weight of 3.4 × 10^6^
SL1: Surfactant, a hydroxyl sulfobetaine, with a purity of 33 %
PV: Injection pore volume
